# A TECHNOLOGY-BASED SUPPORT PROGRAM FOR ASIAN AMERICAN FAMILY CAREGIVERS OF PEOPLE LIVING WITH ALZHEIMER’S DISEASE

**DOI:** 10.1093/geroni/igae098.2736

**Published:** 2024-12-31

**Authors:** Dongmi Kim, Eun-Ok Im, Wonshik Chee, TACAD Research Team

**Affiliations:** The University of Texas Austin, Austin, Texas, United States; The University of Texas Austin, Austin, Texas, United States; The University of Texas Austin, Austin, Texas, United States; The University of Texas Austin, Austin, Texas, United States

## Abstract

The negative impact of family caregiving on health outcomes of caregivers of persons living with Alzheimer’s Disease (PLAD) has been well documented. Especially, Asian American midlife women who are PLAD (AWPLAD) reportedly suffer from caregiving burden because of their cultural attitudes related to Alzheimer’s Disease and caregiving. The first technology-based culturally tailored information and coaching/support program (TACAD) was developed while considering their cultural attitudes. This is a part of a larger 2-phase, mixed-methods, exploratory study to preliminarily evaluate TACAD. Only the first phase is included in this presentation and the 2nd phase is ongoing. The first phase included an expert review and a usability test. The expert review was conducted among 5 experts in family caregivers of PLAD, and a usability test was conducted among 11 AWPLAD. The qualitative data from the expert review and usability text were analyzed using a content analysis. The expert review indicated the necessity of updates on the education modules with new information; shortening each educational module to have up to 10 slides; eliminating redundancies in the content; strengthening the cultural content; and changing the pictures to those with Asians. The usability test indicated the necessity of including more video clippings related to Alzheimer’s Disease and family caregiving; refining chat functions; and enlarging the font size. All the participants agreed that the program would be helpful and useful for AWPLAD and the program was well-organized and easy to follow. Further studies are warranted on culturally tailored technology-based interventions for this specific population.

